# Prevalence, antimicrobial susceptibility, serotyping and virulence genes screening of *Listeria monocytogenes* strains at a tertiary care hospital in Tehran, Iran

**Published:** 2018-10

**Authors:** Siamak Heidarzadeh, Mohammad Mehdi Soltan Dallal, Mohammad Reza Pourmand, Reihaneh Pirjani, Abbas Rahimi Foroushani, Matina Noori, Aida Babazadeh Naseri

**Affiliations:** 1 Department of Pathobiology, School of Public Health, Tehran University of Medical Sciences, Tehran, Iran; 2 Department of Microbiology and Virology, Zanjan University of Medical Sciences, Zanjan, Iran; 3 Food Microbiology Research Center, Tehran University of Medical Sciences, Tehran, Iran; 4 Department of Obstetrics & Gynecology, Arash Hospital, Tehran University of Medical Sciences, Tehran, Iran; 5 Department of Epidemiology and Biostatistics, School of Public Health, Tehran University of Medical Sciences, Tehran, Iran

**Keywords:** *Listeria monocytogenes*, Pregnant women, Antimicrobial susceptibility, Serotyping, Virulence genes

## Abstract

**Background and Objectives:**

*Listeria monocytogenes* is the etiological agent of listeriosis, a highly fatal infection which causes miscarriage or stillbirth in pregnant women. The objective of this study was to detect the prevalence, serotypes, antimicrobial susceptibility and virulence factors of *L. monocytogenes* isolated from pregnant women with vaginitis at a tertiary care hospital in Tehran, Iran.

**Materials and Methods:**

During September 2015 to February 2017, a total of 400 vaginal swabs were collected from pregnant women. The presumptive isolates were characterized biochemically. All *L. monocytogenes* isolates were further analyzed by serotyping and antimicrobial susceptibility tests. All positive samples for *L. monocytogenes* were analyzed for presence of virulence genes (*hly*A, *act*A, *inl*A, *inl*C, *inl*J and *prf*A).

**Results:**

Twenty-two (5.5%) of the samples were found positive for presence of *L. monocytogenes.* Most isolates are resistant to trimethoprim/sulfamethoxazole (81.82%) and chloramphenicol (54.55%). The majority of tested isolates (59.10%) belonged to serotype 4b, followed by 1/2a (22.73%), 1/2b (13.63%), and 3c (4.54%). The *hly*A, *act*A and *inl*A were detected in all of the 22 *L. monocytogenes* isolates, but two, three and five isolates were found to lack *inl*C, *inl*J and *prf*A, respectively. Only one isolate lacked three *inl*C, *inl*J and *prf*A genes, and two isolates simultaneously lacked both *inl*J and *prf*A genes.

**Conclusion:**

Evaluation of virulence factors and antimicrobial susceptibility can be highly helpful to develop effective treatment strategies against *L. monocytogenes* infections. This study is noteworthy in that it documents prevalence, virulence characteristics, and antimicrobial resistance of *L. monocytogenes*.

## INTRODUCTION

*Listeria monocytogenes* is a foodborne pathogen that can cause life-threatening disease in fetuses, newborns, elderly and immunocompromised people (1). It has been stated that pregnant women account for 20–30% of listeriosis cases and listeriosis in pregnant women can lead to bacteremia, amnionitis and infection of the fetus, resulting in premature delivery, miscarriage, stillbirth and other serious health problems for neonates (2, 3). Listeriosis has a mortality rate of about 20% (3).

*L. monocytogenes* includes a spectrum of strains with a wide variation in virulence and pathogenicity. Although the numerous strains of *L. monocytogenes* are naturally virulent and capable of producing high morbidity and mortality, others are non-virulent and unable to cause an infection within hosts (4). Distinction between virulent and avirulent strains is of great importance in assessing the potential implications of these bacteria in food safety and public health (5).

*L. monocytogenes* infection is mediated by many virulence factors. Diverse *Listeria* determinants, which are well known as important factors in the pathogenicity of *L. monocytogenes*, include listeriolysin O (encoded by *hly*A gene), actin (encoded by *act*A gene), internalins (encoded by *inl*A, *inl*C and *inl*J genes) and virulence regulator (encoded by *prf*A gene) (6). The quick and reliable diagnosis of listeriosis has been recommended to be preferably based on the recognition of virulence determinants of *L. monocytogenes* via molecular techniques (7). The objectives of the present study included the detection and characterization of *L. monocytogenes* using cultural and biochemical tests, antimicrobial susceptibility, serotyping and survey of its *hly*A, *inl*A, *inl*C, *inl*J, *act*A and *prf*A virulence genes in isolates obtained from pregnant women using conventional and molecular methods.

## MATERIALS AND METHODS

### Samples.

During September 2015 to February 2017, a total of 400 vaginal swabs were collected from pregnant women with vaginitis. These women had a complicated obstetric history like spontaneous and repeated abortions, stillbirths, pre-term labor and were hospitalized at a tertiary care hospital in Tehran, Iran.

### Ethical approval.

The study was approved by the Ethics Committee of Tehran University of Medical Sciences, number IR.TUMS.SPH.REC.1395.1485.

### Isolation and identification.

Initially, the specimens were inoculated in Buffered *Listeria* Enrichment Broth (BLEB, Merck, Germany) and were incubated at 4°C for 2 weeks to 1 month. The inoculum was then plated on PALCAM agar (Merck, Germany), Oxford agar (Difco, USA) and CHROM agar *Listeria* (Paris, France) plates. After 48 h of incubation at 37°C, colonies morphologically resembling *Listeria* were submitted for confirmatory examinations using Gram staining, catalase and oxidase tests, motility and sugar fermentation tests (xylose, rhamnose, mannitol, α-methyl D-mannopyranoside), hemolysis on 5% sheep blood agar and CAMP test (8, 9). In CAMP test, the *L. monocytogenes* isolates were streaked perpendicular to *Staphylococcus aureus* on 5% sheep blood agar plates and zones of hemolysis were investigated, after 24–48 h of incubation at 35°C (10).

### Serotyping.

All of the *L. monocytogenes* isolates were serotyped by somatic (O) and flagellar (H) antigens specific antisera (Denka Seiken, Tokyo, Japan) (11).

### Antibiotics susceptibility testing.

Fresh bacterial colonies of *L. monocytogenes* isolates were separately grown at 37°C in brain heart infusion broth (BHI, Merck, Germany) for 24 hours and each inoculum was applied on Mueller Hinton Agar with 5% Sheep Blood (Merck, Germany) (12). Susceptibility to a panel of 10 antibiotics (ampicillin 25 μg, gentamicin 10 μg, penicillin G 10 μg, trimethoprim 5 μg, doxycycline 30 μg, ciprofloxacin 5 μg, sulfamethoxazole 25 μg, erythromycin 15 μg, streptomycin 25 μg and chloramphenicol 30 μg) (MAST, UK) was determined using the standard disk diffusion Kirby-Bauer method (13). The inhibition zone diameters (IZD) were interpreted according to CLSI standards for *S. aureus* ATCC 25923 due to lack of specific standards for *Listeria* species (14).

### Molecular detection of virulence genes.

Genomic DNA was isolated from pure cultures of the selected *L. monocytogenes* strains using Qiagen RNA/DNA Kits (Qiagen, USA). All isolates were screened for the *hly*A, *inl*A, *inl*C, *inl*J, *act*A and *prf*A genes. The primers described by Liu et al. (2007), and Nayak et al. (2015) were used for detection of *inl*A/C/J, and *act*A, respectively (4, 15). Also, the *hly*A and *prf*A primers were designed in this study (Table 1). The PCR mixture contained 12.5-μL mastermix PCR, 1 μL of each primer, and 50 ng of DNA in a 25-μL final volume. PCR amplification was performed in a thermal cycler instrument (MJ Research Inc., MA, USA) and included initial denaturation at 94°C for 5 min, and then subjected to 30 cycles of amplification (denaturation at 95°C for 1 min, annealing at primer-specific temperature for 30–60 s, and extension at 72°C for 30 s) followed by a final extension step at 72°C for 10 min. Amplicons were separated via gel electrophoresis (70 min at 90 V) on 1% agarose 0.5 X TBE buffer and visualized under UV light after staining with ethidium bromide.

**Table 1.** Identities and nucleotide sequences of *L. monocytogenes* virulence gene primers

**PCR test**	**Primer name**	**Sequence (53→′)**	**Target gene**	**Annealing temperature**	**Size of amplicon**	**Ref**
mPCR1	*inl*A-F	ACGAGTAACGGGACAAATGC	*inl*A	60°C	800 bp	Liu
	*inl*A-R	CCCGACAGTGGTGCTAGATT				
	*inl*C-F	AATTCCCACAGGACACAACC	*inl*C		517 bp	Liu
	*inl*C-R	CGGGAATGCAATTTTTCACTA				
	*inl*J-F	TGTAACCCCGCTTACACAGTT	*inl*J		238 bp	Liu
	*inl*J-R	AGCGGCTTGGCAGTCTAATA				

PCR2	*prf*A-F	GACCGCAAATAGAGCCAAGC	*prf*A	60°C	181 bp	This study
	*prf*A-R	GAAGTCATTAGCGAGCAGGC				

PCR3	*hly*A-F	GCGCAACAAACTGAAGCAAA	*hly*A	60°C	221 bp	This study
	*hly*A-R	TAACCTTTTCTTGGCGGCAC				

PCR4	*act*A-F	ACCGCCTCCAACAGAAGATG	*act*A	56°C	644 bp	Nayak
	*act*A-R	GGATTACTGGTAGGCTCGGC				

### Statistical analysis.

All data were collected and analysis was done using SPSS version 23 and for survey of significance, Chi-square test was calculated. A value of p ≤ 0.05 was also considered statistically significant.

## RESULTS

A total of 400 samples were screened for the presence of *L. monocytogenes*. Twenty-two (5.5%) of the samples were found positive for the presence of *L. monocytogenes*. All the 22 isolates showed characteristic enhancement of the hemolytic zone with *S. aureus* in the CAMP test.

In this study, the percentage of isolates resistant to antibiotics was found as follows: penicillin G 45.45%, gentamicin 36.36%, ampicillin 45.45%, trimethoprim 81.82%, tetracycline 45.45%, ciprofloxacin 18.18%, sulfamethoxazole 81.82%, erythromycin 45.45%, streptomycin 45.45%, and chloramphenicol 54.55%. The majority of isolates were resistant to trimetho-prim/sulfamethoxazole, whereas the lowest resistance was shown to ciprofloxacin.

In total, all the *L. monocytogenes* isolates were resistant to three or more antimicrobial agents. Among the resistant isolates, two, five, nine and three isolates, respectively, were resistant to three, four, five and six antibiotics. Also, one isolate was resistant to 8 antibiotics and one isolate was resistant to 9 antibiotics. Surprisingly, an isolate was resistant to all antimicrobials.

All isolates resistant to penicillin G, ampicillin, tetracycline, erythromycin, and chloramphenicol belonged to serotypes 4b, 1/2a and 1/2b, while isolates resistant to ciprofloxacin, gentamicin, streptomycin and trimethoprim/sulfamethoxazole belonged to serotypes 4b, 1/2a, 1/2b and 3c.

The majority of tested isolates (13, 59.10%) belonged to serotype 4b, followed by 1/2a (5, 22.73%), 1/2b (3, 13.63%) and 3c (1, 4.54%) (Table 1).

**Fig. 1.**
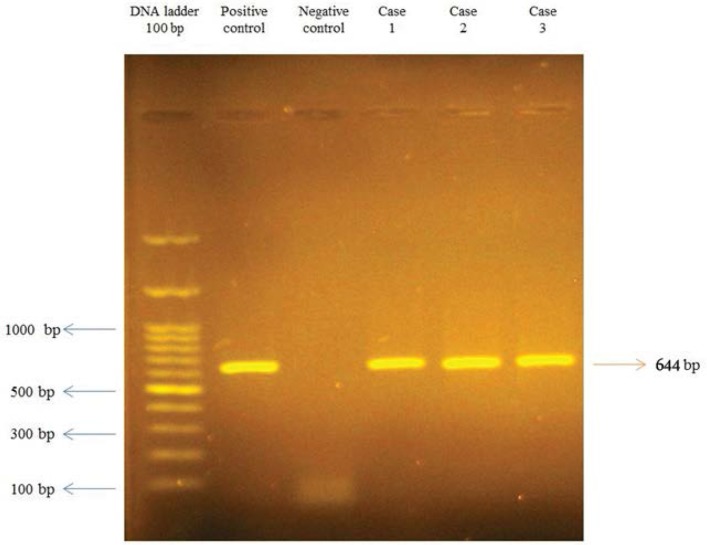
Polymerase chain reaction (PCR) analyses of *act*A gene. Agarose gel electrophoresis of the 644-bp fragments of the *act*A gene.

**Fig. 2.**
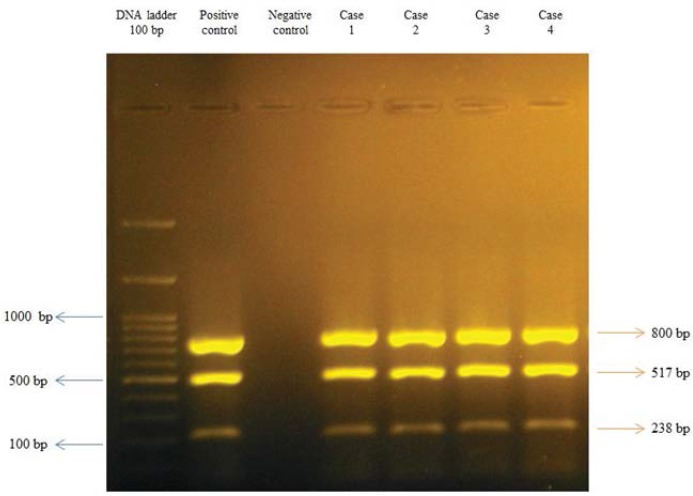
Polymerase chain reaction (PCR) analyses of *inl*A/C/J genes. Agarose gel electrophoresis of the 800, 517, 238-bp fragments of the *inl*A/C/J genes, respectively.

Twenty-two isolates of *L. monocytogenes* obtained from vaginal samples were screened for the presence of *hly*A, *act*A, *inl*A, *inl*C, *inl*J and *prf*A genes. The *hly*A, *act*A and *inl*A genes were detected in all the 22 *L. monocytogenes* isolates (Fig. 1), but two, three and five isolates were found to lack *inl*C, *inl*J (Fig. 2) and *prf*A, respectively. Only one isolate simultaneously lacked three *inl*C, *inl*J and *prf*A genes, also two isolates lacked both *inl*J and *prf*A genes (Table 2).

**Table 2.** Serotypes and virulence genes in clinical isolates

**Code**	***hly*A**	***act*A**	***inl*A**	***inl*C**	***inl*J**	***prf*A**	**Serotypes**
1	+	+	+	+	+	+	4b
2	+	+	+	+	+	−	1/2b
3	+	+	+	+	+	+	4b
4	+	+	+	+	+	+	4b
5	+	+	+	+	+	+	4b
6	+	+	+	−	−	−	3c
7	+	+	+	+	+	+	4b
8	+	+	+	+	+	+	4b
9	+	+	+	−	+	+	1/2a
10	+	+	+	+	+	−	1/2a
11	+	+	+	+	+	+	1/2b
12	+	+	+	+	+	+	4b
13	+	+	+	+	−	−	1/2a
14	+	+	+	+	+	+	4b
15	+	+	+	+	+	+	4b
16	+	+	+	+	+	+	4b
17	+	+	+	+	+	+	4b
18	+	+	+	+	−	−	1/2a
19	+	+	+	+	+	+	1/2b
20	+	+	+	+	+	+	1/2a
21	+	+	+	+	+	+	4b
22	+	+	+	+	+	+	4b

## DISCUSSION

Serotyping is an additional effective tool for identifying *L. monocytogenes* isolates (16). Although most clinical isolates belong to serotype 4b, the majority of food isolates belong to serotype 1/2a or 1/2b. Thus, it is likely that serotype designation is related to virulence potential (17). The majority of tested isolates (13, 59.10%) belonged to serotype 4b, followed by 1/2a, 1/2b and 3c.

Recently, there have been reports of increased resistance to most commonly used antibiotics among *L. monocytogenes* strains, causing serious problems in the management of human listeriosis cases. The multidrug resistance (MDR) *L. monocytogenes* related to human listeriosis has been described from food and the environment (18). Some studies conducted in Iran have described the resistance of *L. monocytogenes* to tetracycline, penicillin G, streptomycin, sulfamethoxazole, gentamycin, erythromycin, and ciprofloxacin (19). Dehkordi et al. (20), Rahimi et al. (21), and Jamali et al. (6) isolated MDR *L. monocytogenes* from veterinary, food, environmental and clinical samples. Like other studies, the present study showed that most isolates of *L. monocytogenes* are resistant to three or more antibiotics.

Instant isolation and confirmation techniques for *L. monocytogenes* are still required. Some non-pathogenic strains behave phenotypically closely related to pathogen strains (22), and many strains of *L. monocytogenes* are different in pathogenic potential and virulence (23). A number of *L. monocytogenes* strains are naturally virulent yielding high morbidity and mortality, while others which are avirulent produce no obvious disease (24). PCR-based tests for the key virulence-associated genes yield quick and reproducible results (18, 25). In a study by Eslami et al. 16.7% of samples tested had been positive for *L. monocytogenes* (26). In Sadeghi Kalani’s study, the incidence of *L. monocytogenes* in clinical samples was reported as 8.23% (27). In a study conducted by Jahangirsisakht et al. in Iran, out of 107 samples, *L. monocytogenes hly*A gene was detected in 11 samples (10.28%) (28). However, in Iran, few studies have evaluated the prevalence, identification of virulent and non-virulent strains, as well as virulence factors of *L. monocytogenes* isolates in clinical samples. Stepanović et al. (2007) reported low frequency of *L. monocytogenes* in clinical samples (0.1%) (29). In another study, Soni et al. (2015) isolated *L. monocytogenes* from 0.81% of clinical samples (30). However, in Egal et al.’s study (2015), the incidence of *L. monocytogenes*-associated abortion and stillbirth was from 0 to 8.39% through out 1989 to 2009 (31). Also, Shindang et al. (2013) isolated *L. monocytogenes* from 8.04% of blood and placenta samples (32). In a study carried out in India by Kaur et al. in 2007 on spontaneous abortion, they isolated *Listeria* spp. and *L. monocytogenes* from 14.8% and 3.3% of specimens, respectively. In the research, they also studied *plc*A, *prf*A, *act*A, *hly*A and *ia*p genes (33).

Probably, the best results were achieved through evaluation of several genes; therefore, it is recommended that numerous major virulence factors of *L. monocytogenes* should be investigated.

## CONCLUSION

In Iran, the real prevalence of *L. monocytogenes* is indefinite and only few studies have been conducted on listeriosis. Moreover, listeriosis is not a reportable disease in the Iranian health system. Therefore, further attention and studies are required to investigate and determine accurate listeriosis status in Iran. Regarding the high sensitivity and specificity of molecular techniques, we suggest to use these methods for the identification of virulence genes and also differentiate between virulent and avirulent strains of *L. monocytogenes*. In conclusion, the evaluation of virulence factors and antimicrobial susceptibility can be highly helpful in development of effective treatment strategies against *L. monocytogenes* infections.
